# Clinical, pathological, and molecular features of classical and L-type atypical-BSE in goats

**DOI:** 10.1371/journal.pone.0198037

**Published:** 2018-05-24

**Authors:** Elena Vallino Costassa, Antonio D’Angelo, Maria Mazza, Daniela Meloni, Elisa Baioni, Cristiana Maurella, Silvia Colussi, Nicola Martinelli, Monica Lo Faro, Elena Berrone, Alessandra Favole, Paola Crociara, Silvia Grifoni, Marina Gallo, Guerino Lombardi, Barbara Iulini, Cristina Casalone, Cristiano Corona

**Affiliations:** 1 Centre of Animal Encephalopathies (CEA), Istituto Zooprofilattico Sperimentale del Piemonte, Liguria e Valle d’Aosta, Turin, Italy; 2 Dipartimento di Scienze Veterinarie, Sezione Clinica Medica, University of Turin, Grugliasco (Turin), Italy; 3 Istituto Zooprofilattico Sperimentale della Lombardia e dell'Emilia Romagna, Brescia, Italy; Van Andel Institute, UNITED STATES

## Abstract

Monitoring of small ruminants for transmissible spongiform encephalopathies (TSEs) has recently become more relevant after two natural scrapie suspected cases of goats were found to be positive for classical BSE (C-BSE). C-BSE probably established itself in this species unrecognized, undermining disease control measures. This opens the possibility that TSEs in goats may remain an animal source for human prion diseases. Currently, there are no data regarding the natural presence of the atypical BSE in caprines. Here we report that C-BSE and L-type atypical BSE (L-BSE) isolates from bovine species are intracerebrally transmissible to goats, with a 100% attack rate and a significantly shorter incubation period and survival time after C-BSE than after L-BSE experimental infection, suggesting a lower species barrier for classical agentin goat. All animals showed nearly the same clinical features of disease characterized by skin lesions, including broken hair and alopecia, and abnormal mental status. Histology and immunohistochemistry showed several differences between C-BSE and L-BSE infection, allowing discrimination between the two different strains. The lymphoreticular involvement we observed in the C-BSE positive goats argues in favour of a peripheral distribution of PrPSc similar to classical scrapie. Western blot and other currently approved screening tests detected both strains in the goats and were able to classify negative control animals. These data demonstrate that active surveillance of small ruminants, as applied to fallen stock and/or healthy slaughter populations in European countries, is able to correctly identify and classify classical and L-BSE and ultimately protect public health.

## Introduction

Many mammalian species can be affected by prion diseases or transmissible spongiform encephalopathies (TSEs), fatal neurodegenerative diseases caused by the conformational conversion of the normal, host-encoded cellular prion protein (PrPC) into a pathological protease-resistant isoform, termed pathological prion protein (PrPSc). TSEs include bovine spongiform encephalopathy (BSE) in cattle, scrapie in sheep and goats, chronic wasting disease (CWD) in cervids, transmissible mink encephalopathy (TME) in mink and Kuru, and Creutzfeldt-Jakob disease (CJD) in humans.

“Classical” BSE (C-BSE) was the first prion disease recognized in cattle. Epidemiological studies suggest that ruminant-derived meat and bone meal (MBM) containing central nervous system (CNS) tissue contaminated with PrPSc was the source of the outbreak in the United Kingdom (UK) [1]. Since 2004, two atypical strains of BSEs have been distinguished from the classical type, based on a higher or lower apparent molecular mass profile of the unglycosylated PrPSc band: H-BSE and L-BSE, respectively, or bovine amyloidotic spongiform encephalopathy (BASE) [2,3]. Given the epidemiological characteristics of these forms, it has been speculated that the atypical strains are genetic in origin and/or arise spontaneously.

The spread of the BSE agent in small ruminants has been considered a major threat in recent years because sheep and goats were exposed to the same contaminated feedstuffs as cattle during the UK BSE epizootic. To date, there are no reports of naturally occurring C-BSE in sheep, whereas two cases in goats were confirmed in France and the UK [4,5]. According to the data from these studies, the PrPSc distribution is limited to the brain in these natural cases. Experimental studies to better characterize the C-BSE phenotype in the event that it is transmitted to sheep and goats have shown that both species are readily infected with the agent of C-BSE [6,7,8] and that PrPSc distribution is very similar to that observed in classical scrapie, resulting in infectivity throughout lymphoid tissues [6,9] with some interspecies variability.

Our current knowledge of atypical strains in small ruminants relies on experimental challenges in sheep. L-BSE was successfully transmitted via the intracerebral route to homozygous A_136_R_154_Q_171_ sheep that developed terminal disease at 29±3 months post inoculation (m.p.i.) and PrPSc accumulation only in the CNS [10]. Other studies reported PrPSc deposition in both the central and the peripheral nervous system, without lymphoid tissue involvement, in a ewe intracranially inoculated with cattle L-BSE [11]. Recently, L-BSE was intracerebrally transmitted to sheep of several genotypes, producing a cataplectic form of disease characterized by collapsing episodes and reduced muscle tone, with PrPSc accumulation limited to the nervous system, except for one animal with lymphoreticular involvement [12].

Here we describe, for the first time, the clinical, pathological, and molecular features of L-BSE in goats as compared to goats experimentally challenged with C-BSE. These findings extend our view of the phenotypical differences between prion protein disorders in goat species and challenge current strategies for their diagnosis and classification. Furthermore, possible implications for surveillance and control policies for TSEs in goats are also discussed.

## Results

### Survival analysis

Incubation period (IP) and survival time (SV) (Table 1) were evaluated using Kaplan-Meier survival estimates and log-rank tests.

**Table 1:** Survival time (SV) and incubation period (IP) in C-BSE and L-BSE infected goats and mean SV in negative goats.

Inoculum	Attack rate	Individual SV (dpi)	Mean SV (±SD)	Individual IP (dpi)	Mean IP (±SD)	Mean SV in negative goats (dpi)
**C-BSE i.c.**	4/4	583, 645, 630, 640	624,5 (±28.36)	533, 594, 594, 552	568,25 (±30.72)	659
**L-BSE i.c.**	6/6	1504, 930, 1595, 965, 1352, 1088	1239 (±283.93)	1295, 825,1498, 651, 1260, 959	1081.33 (±321.57)	1481

#### Incubation period

**Fig 1:**
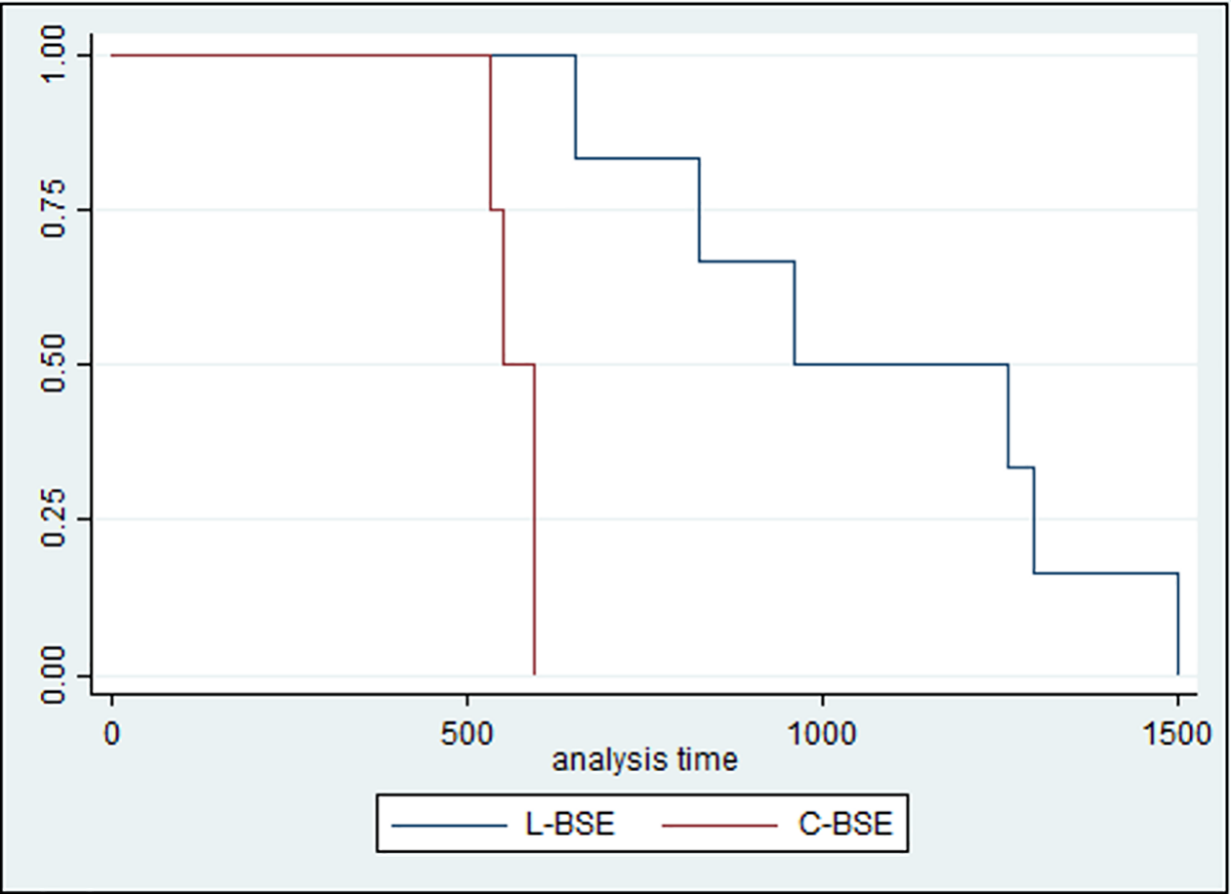
Kaplan–Meier curves for incubation period.

The median follow-up period was 553 days (range, 534 to 595) for the C-BSE inoculated group and 960 days (range, 652 to 1499) for the L-BSE inoculated group; the average hazard rate was 0.002 and 0.0009, respectively. The analysis revealed a statistically significant difference in incubation period between the two groups (p = 0.002) (Fig 1).

#### Survival time

**Fig 2:**
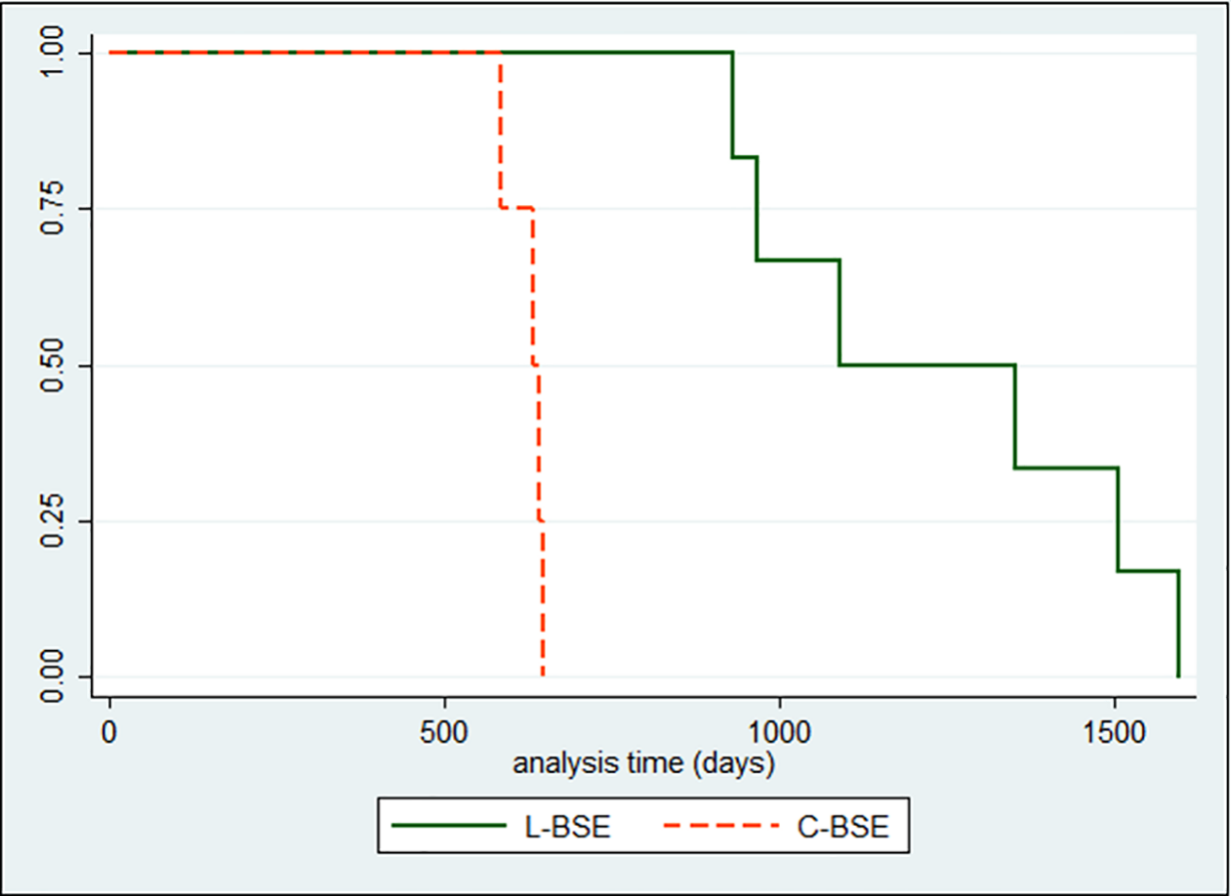
Kaplan–Meier survival curves for survival times.

The median duration of follow-up was 631 days (range, 584 to 646) for the C-BSE inoculated group and 1089 days (range, 931 to 1596) for the L-BSE inoculated group; the average hazard rate was 0.002 and 0.0008, respectively. The difference in survival time between the two groups was statistically significant (p = 0.001) (Fig 2).

### Clinical features

All the animals intracerebrally inoculated with either C-BSE or L-BSE had ARQ/ARQ genotype with codon 240 P/P, except for 2 goats with codon 240 S/P. They developed neurological signs and were euthanized at the terminal stage of disease. Clinical signs were not observed in control animals and they were euthanized at 659 (C-BSE) and 1481 (L-BSE) dpi (mean survival time). In the C-BSE inoculated goats, the early clinical signs included skin lesions on the neck, shoulders, sacral region, hind limbs, and tail and were characterized by broken hair and alopecia caused by scratching. As the disease progressed, the animals displayed depressed mental status, disorientation, ataxia, paresis, abnormal proprioception/postural reactions, decreased menace reaction, and positive nibble reflex. The early clinical presentation in the goats inoculated with L-BSE was characterized by skin lesions with alopecia and broken hair, generalized tremors, and hind limb lameness. Except for positional strabismus in 1 animal and positional vertical nystagmus in 2 animals, all goats subsequently developed neurological signs similar to those described for the C-BSE inoculated goats (Table 2).

**Table 2:** Number of animals presenting with clinical signs.

Inoculum	Skin Lesion	Decreased body condition score	Abnormal Mental Status	Abnormal Behaviour	Abnormal Posture	Abnormal Gait	Proprioceptionand Postural Reaction Deficits	Cranial Nerve Deficits	Positive Nibble Reflex
**C-BSE** **(*n* = 4)**	4	1	4	4	3	3	3	3	3
**L-BSE** **(*n* = 6)**	6	2	3	3	5	5	2	5	5

### Pathology and immunohistochemistry

**Fig 3:**
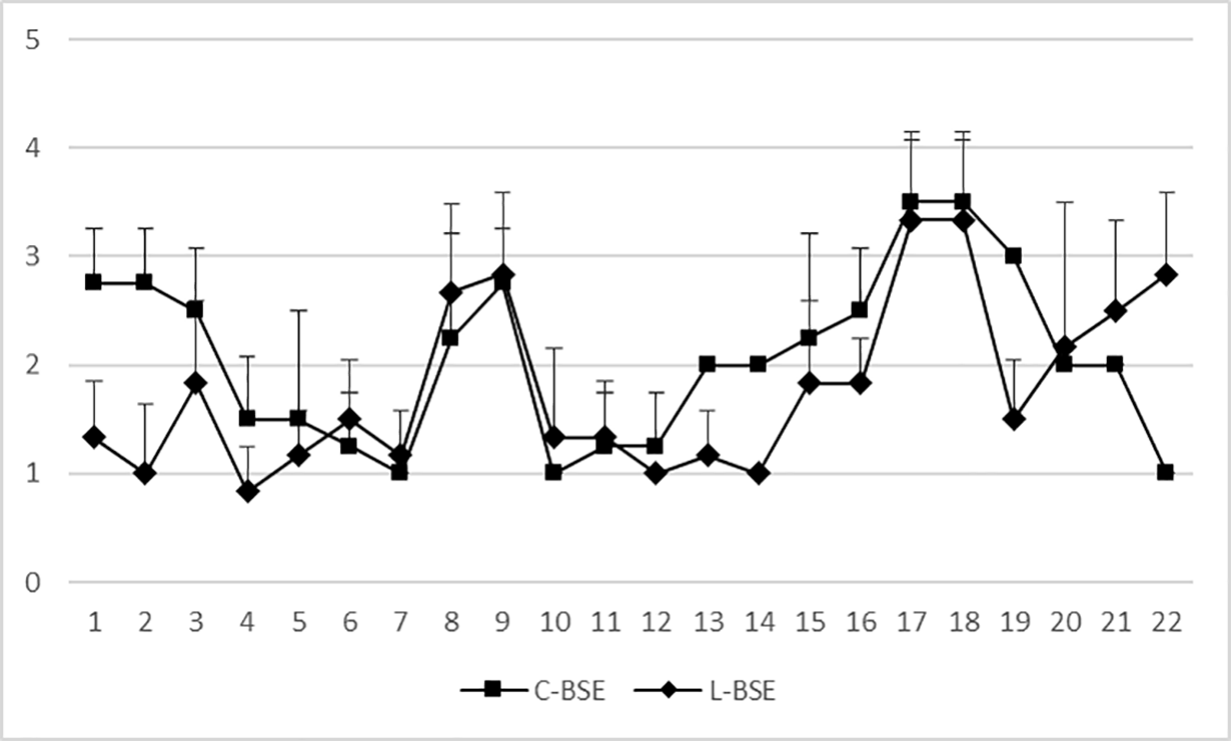
Mean values of vacuolar lesion profiles. Square = BSE i.c. goats; rhombus = L-BSE i.c. goats (Brain areas, Medulla (at obex): 1 Dorsal nucleus of the vagus nerve, 2 Nucleus of the hypoglossal nerve, 3 Reticular formation, 4 Midline Raphe, 5 Accessory cuneate nucleus, 6 Olivary nuclei; Rostral medulla: 7 Vestibular nuclear complex, 8 Cochlear nucleus, 9 Nucleus of the spinal tract of the trigeminal nerve, 10 Midline raphe; Cerebellar vermis: 11 Nodulus±granular layer, 12 Nodulus±molecular layer; Midbrain: 13 Central grey matter, 14 Red nucleus, 15 Substantia nigra, 16 Lateral geniculate nucleus; Thalamus: 17 Dorsomedial thalamic nucleus, 18 Ventral thalamic nuclei, 19 Area hypothalamica; Frontal: 20 Caudate nucleus, 21 Nucleus accumbens, 22 Frontal cortex).X-axis: brain areas; Y-axis: mean vacuolation score with error bars (standard deviation).

**Fig 4:**
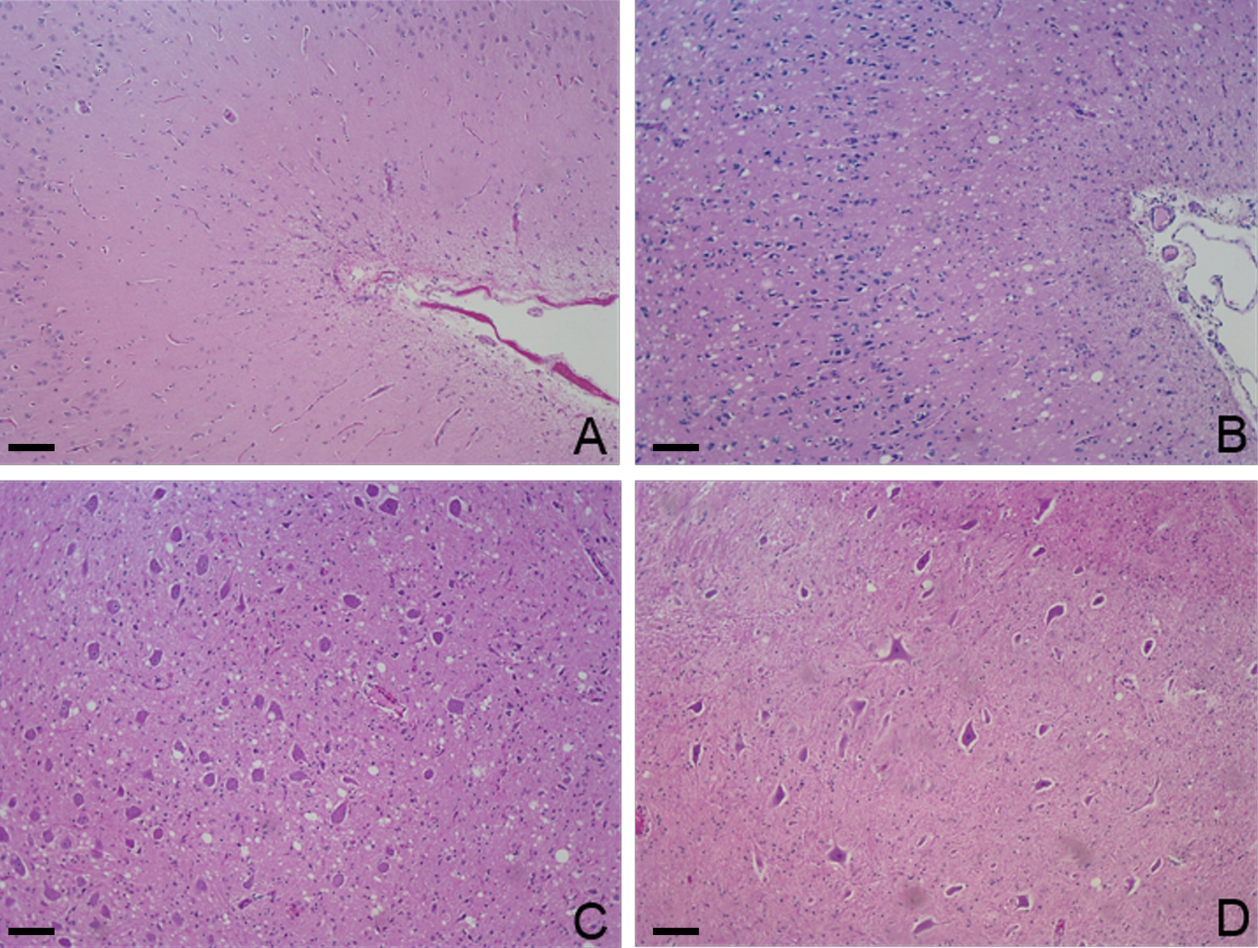
Haematoxylin and eosin. A: Frontal cortex of C-BSE (10X); B: Frontal cortex of L-BSE (10X); C: Dorsal nucleus of vagus nerve, brainstem of C-BSE (10X); D: Dorsal nucleus of vagus nerve, brainstem of L-BSE (10X). The single image shows one animal (81556 for C-BSE and 69540 for L-BSE) representative of all animals in the group. Scale bar: 100 μm.

The overall intensity of neuropil vacuolation was generally similar in all the animals that had received the same inoculum and differed only partially between the two groups. Intense spongiosis of the caudal regions (brainstem and medulla) was observed in the C-BSE group, whereas vacuolation of the caudate and accumbens nuclei and the frontal cortex was greater in the L-BSE group. The thalamus and the nucleus of the spinal tract of the trigeminal nerve in the rostral medulla, were involved in both groups (Figs 3 and 4).

**Fig 5:**
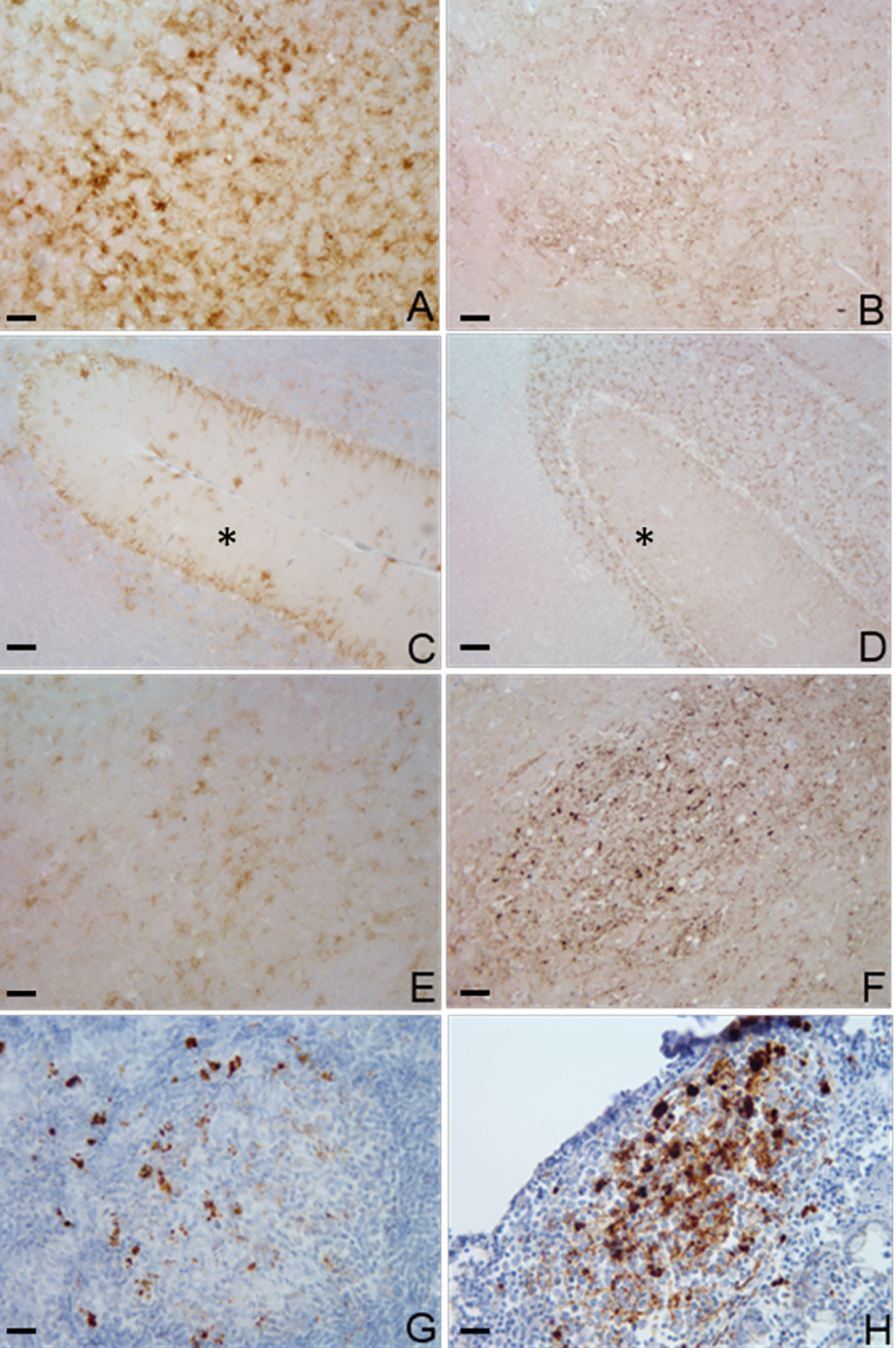
Immunohistochemistry. Labelling with F99/97.6.1, with DAB used as chromogen; the immunoreactivity is brown in colour. A: Brainstem, reticular formation, C-BSE (10X); B: Brainstem, reticular formation, L-BSE (10X); C: Cerebellum, C-BSE (10X), *: molecular layer; D: Cerebellum, L-BSE (10X), *:molecular layer; E: Midbrain, central grey matter, C-BSE (10X); F: Midbrain, substantia nigra, L-BSE (10X); Scale bar: 100 μm. G: Submandibular lymph node, C-BSE (40X); H: Ileocecal valve, C-BSE (40X); Scale bar: 25 μm.

**Fig 6:**
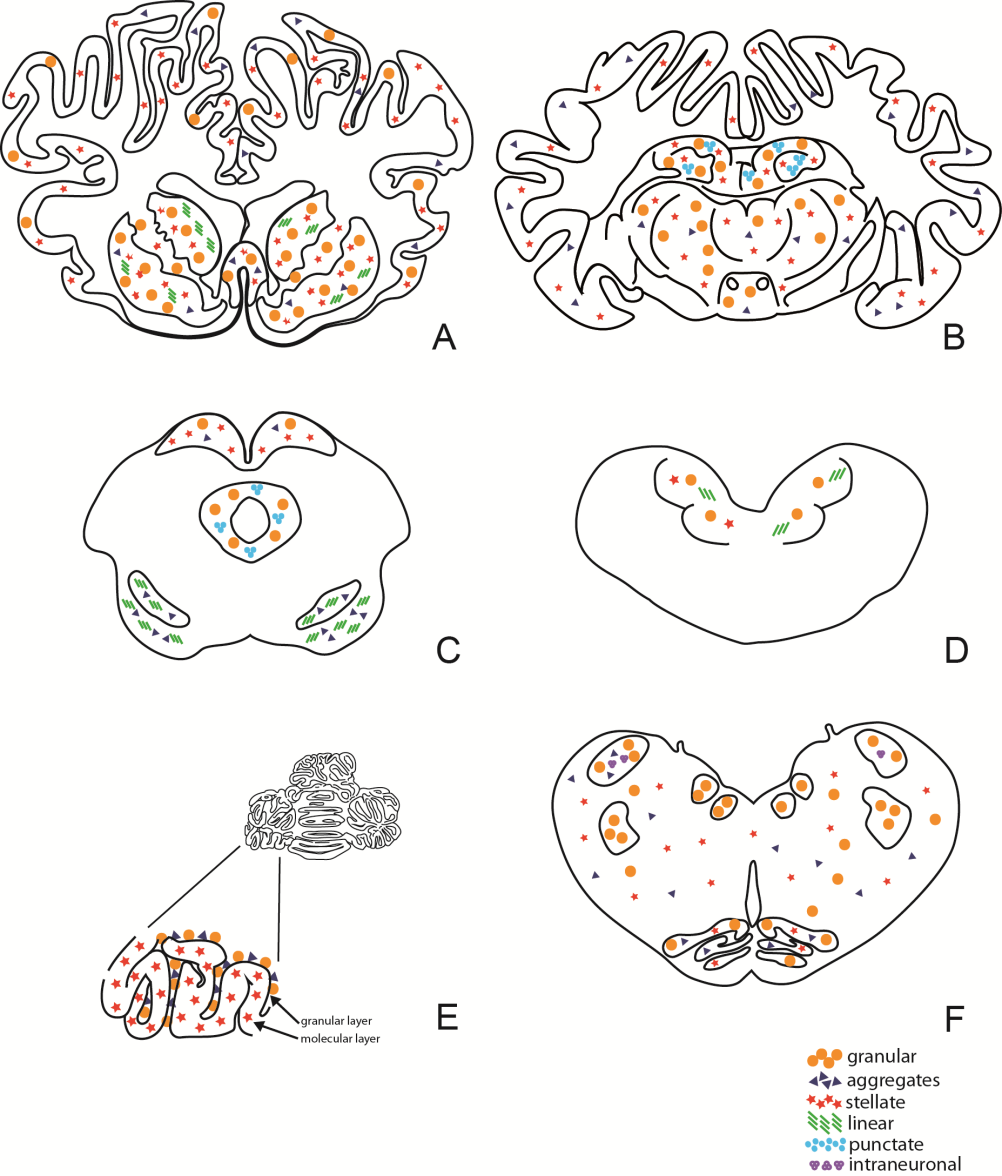
Non-quantitative diagram of neuroanatomical distribution of PrP immunolabelling in the C-BSE group. This distribution refers to one animal (81556) representative of all animals in the group. A: Telencephalon; B: Diencephalon; C: Mesencephalon; D: Pons; E: Cerebellum; F: Brainstem.

**Fig 7:**
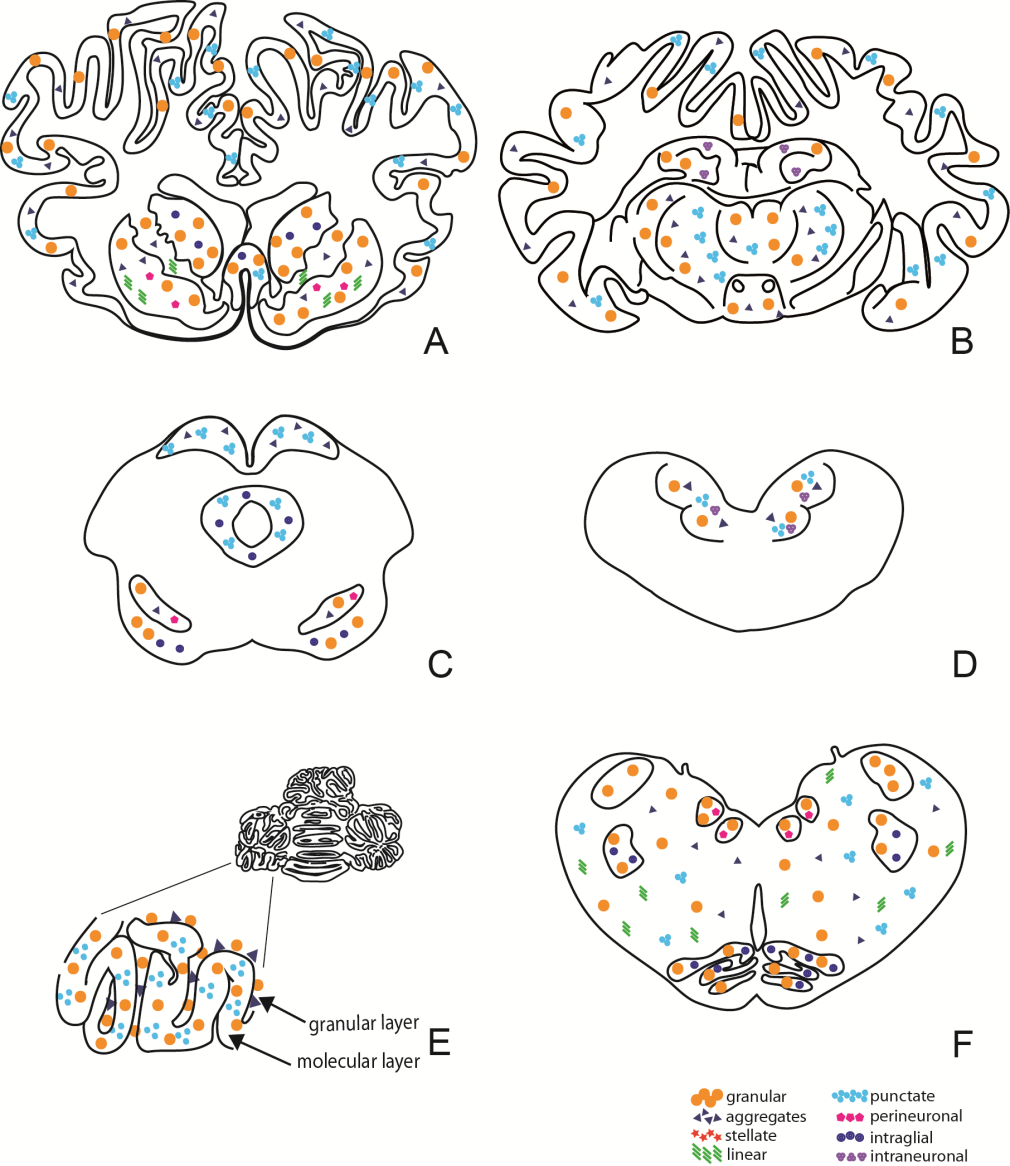
Non-quantitative diagram of neuroanatomical distribution of PrP immunolabelling in L-BSE group. This distribution refers to one animal (69540) representative of all animals in the group. A: Telencephalon; B: Diencephalon; C: Mesencephalon; D: Pons; E: Cerebellum; F: Brainstem.

Widespread immunolabelling with monoclonal antibody (mAb) F99/97.6.1 was present at all levels of the brain. Different PrPSc types were identified and were very similar to those described by González et al. [13]. Coarse particulate and stellate PrPSc deposition patterns were mainly observed in several brain areas in the C-BSE inoculated goats, including the frontal cortex, basal ganglia, thalamus, hypothalamus, midbrain, pons, cerebellum, and brainstem. In the L-BSE inoculated goats, these same areas mainly displayed a coarse particulate and a fine punctate pattern. Linear tracts, intraglial, intraneuronal, perineuronal, and small aggregates were noted in both groups. Small aggregates sometimes showed a tendency to form small, coreless plaques that were more often present in the C-BSE than in the L-BSE group. There was a clear difference in the molecular layer of the cerebellum between the two groups: a stellate pattern in the C-BSE group and a granular pattern in the L-BSE group (Figs 5, 6 and 7).

Using the epitope mapping approach on the same brain areas, in order to discriminate C-BSE and L-BSE from scrapie, intraneuronal and extraneuronal signals of PrPSc were detected by using antibodies directed to the globular domain or the C-terminal portion of the prion protein, whereas only an extraneuronal signal was detected using N-terminal antibodies (Table 3). In detail, immunolabelling with mAb L42 revealed mostly stellate and coarse particulate patterns in the CNS of the C-BSE challenged goats, mainly at the level of frontal cortex, basal ganglia, thalamus, and midbrain, whereas coarse particulate and small aggregates were predominant in the L-BSE goats. Immunolabelling with mAb 9A2 produced coarse particulate and stellate patterns, particularly in the basal ganglia, midbrain, and brainstem in the C-BSE goats, whereas mAb 9A2 immunolabelling was generally weak in the L-BSE goats, except for small aggregates present mainly in the thalamus and a stellate pattern in the basal ganglia. Immunolabelling with mAb 12B2 and P4 revealed stellate, linear, and coarse particulate patterns of PrPSc in the brain tissue from the C-BSE goats but no intense immunolabelling in the L-BSE goats. Intraneuronal labelling was not observed in either group with P4 and 12B2. Mab 2G11 showed intense immunolabelling with coarse particulate, stellate, and linear patterns in the C-BSE goats and mainly small aggregates of PrPSc in the L-BSE goats.

**Table 3:** Epitope mapping results for goat C-BSE and L-BSE goats.

	Antibodies					
PrPSc localization	P4	9A2	12B2	L42	2G11	F99
**C-BSE: extraneuronal**	+	+	+	+	+	+
**L-BSE: extraneuronal**	+	+	+	+	+	+
**C-BSE: intraneuronal**	-	n.e.*	-	+	+	+
**L-BSE: intraneuronal**	-	n.e.*	-	+	+	+

*n.e.: not evaluable, due to the reaction’s background.

Determination of PrPSc distribution in the C-BSE goats, based on the signal intensity of labelling, revealed similarly high levels of prion protein accumulation in the thalamus, pons, medulla, and basal ganglia and weaker signal intensity in the mesencephalon and cerebellum; similarly, high levels of the prion protein were identified in the pons, brainstem, and cerebellum and weaker signals in the mesencephalon and basal ganglia of the L-BSE goats. Immunohistochemistry with glial fibrillary acidic protein (GFAP) in the same areas evaluated for PrPSc distribution revealed the presence of gliosis in the experimentally infected animals (data not shown).

### Rapid tests and Western blot analysis

All rapid tests, protocols, and conjugate type were able to detect both C-BSE and L-BSE (Table 4). The IDEXX tests resulted in optical density (OD) values with systematic overflow, whereas the Bio-Rad tests, i.e., the sheep & goat kit, showed some quantitative reduction in comparison to the IDEXX tests for both C–BSE and L-BSE samples.

**Table 4:** Normalized rapid test results: IDEXX HerdChek® BSE-scrapie EIA Short and Ultra Short protocol–small ruminant conjugate, IDEXX HerdChek™ BSE- scrapie EIA Short and Ultra Short protocol—bovine conjugate; Bio-Rad TeSeE™ and Bio-Rad Sheep and Goat™.

INOCULUM/CONTROL	IDEXX HerdChek BSE-scrapie EIA				BioRad TeSeE	
** **	**Short protocol** ^				**TeSeE SAP**	
** **	SRB CC		CC			
** **	**NORMALISED**^¿^ **Log MEAN (SD**^†^**)**	**MEAN (SD)**	**NORMALISED Log MEAN (SD)**	**MEAN (SD)**	**NORMALISED Log MEAN (SD)**	**MEAN (SD)**
**C-BSE**	1.27 (±0.00)	4.00 (±0.00)	1.40 (±0.00)	4.00 (±0.00)	1.39 (±0.01)	3.73 (±0.06)
**C-BSE CONTROL**	0.93 (±0.01)	3.20 (±0.00)	1.05 (±0.03)	3.19 (±0.00)	0.94 (±0.09)	0.09 (±0.00)
**L-BSE**	1.27 (±0.00)	3.99 (±0.00)	1.40 (±0.00)	3.99 (±0.00)	1.36 (±0.04)	3.49 (±0.35)
**L-BSE CONTROL**	0.89 (±0.12)	3.21 (±0.02)	1.05 (±0.01)	3.21 (±0.00)	0.88 (±0.02)	2.79 (±0.00)
	**Ultra-Short protocol**^**$**^				**TeSeE Sheep & Goat**	
	SRB-CC*		**CC**°			
** **	**NORMALISED Log MEAN (SD)**	**MEAN (SD)**	**NORMALISED Log MEAN (SD)**	**MEAN (SD)**	**NORMALISED Log MEAN (SD)**	**MEAN (SD)**
**C-BSE**	1.29 (±0.00)	4.00 (±0.00)	1.40 (±0.00)	4.00 (±0.00)	1.21 (±0.01)	3.54 (±0.08)
**C-BSE CONTROL**	0.94 (±0.01)	3.20 (±0.00)	1.04 (±0.02)	3.21 (±0.00)	0.70 (±0.06)	2.78 (±0.00)
**L-BSE**	1.29 (±0.00)	3.99 (±0.00)	1.40 (±0.00)	3.99 (±0.00)	1.07 (±0.16)	2.73 (±0.84)
**L-BSE CONTROL**	0.90 (±0.05)	3.20 (±0.01)	1.04 (±0.05)	3.21 (±0.01)	0.62 (±0.03)	2.24 (±0.00)

The IDEXX HerdChek® BSE-scrapie EIA test has two approved protocols for brain tissue:

^ Short and

^$^Ultra- Short.

The protocols have equivalent performance but varying equipment requirements for decreased assay time.

There are two conjugate concentrates available for the IDEXX HerdChek® BSE-scrapie EIA test depending on the tissue to be tested: Conjugate concentrate (CC): this conjugate is appropriate when testing bovine brain samples, small-ruminant lymph node, and spleen samples; Small-ruminant brain conjugate concentrate (SRB-CC): This conjugate is used when testing small ruminant brain tissue.

The Bio-Rad TeSeE™ tests include a purification kit that allow purification, concentration, and solubilisation of PrPSc from tissue samples obtained from infected animals and a detection kit that uses an immuno-enzymatic technique (sandwich format) with two monoclonal antibodies for the detection of the abnormal prion protein resistant to proteinase K.

The Bio-Rad TeSeE™ Sheep & Goat test is approved as a rapid test for monitoring of TSE in ovines and caprines, while the Bio-Rad TeSeE™ SAP is approved for BSE and scrapie testing programmes on cattle, sheep, and goats.

° Conjugate concentrate (CC)

* Small-ruminant brain conjugate concentrate (SRB-CC)^¿^ Normalised = Log(x/cut-off)

^†^ SD: Standard deviation.

**Fig 8:**
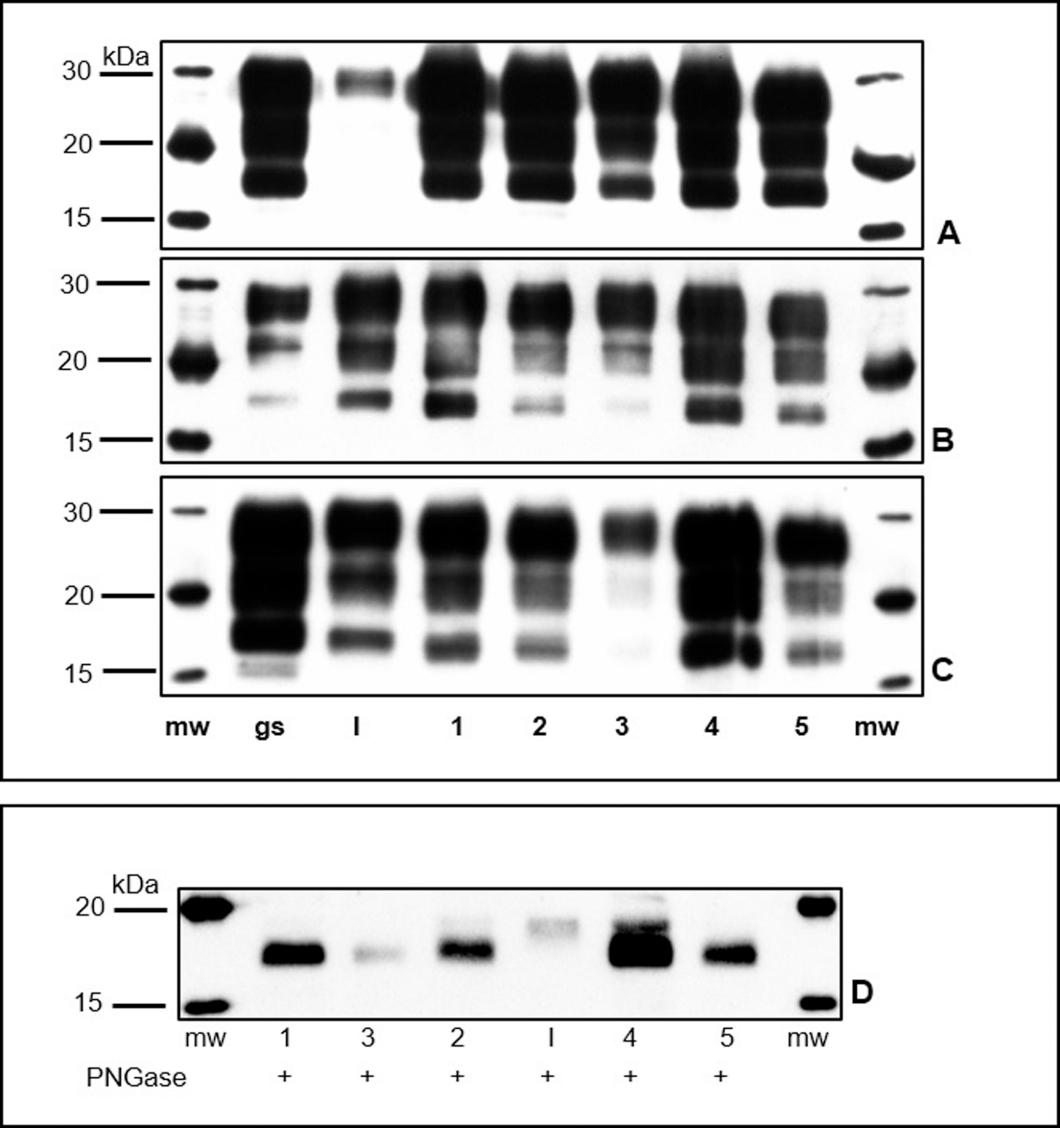
Western blot analysis of proteinase K-treated homogenates from brainstems of goats intracerebrally inoculated with C-BSE. I: C- BSE (16819/06) isolate used for *inoculum*; lanes 1–5: inoculated goats; gs: goat with natural classical scrapie. MW: molecular size markers. Membranes were probed with three different monoclonal antibodies: P4 (A), 6H4 (B), and SAF84 (C). Panel D shows the proteinase K-treated homogenates treated with enzymatic deglycosylation (PNGase) and detected with mAb SAF84.

**Fig 9:**
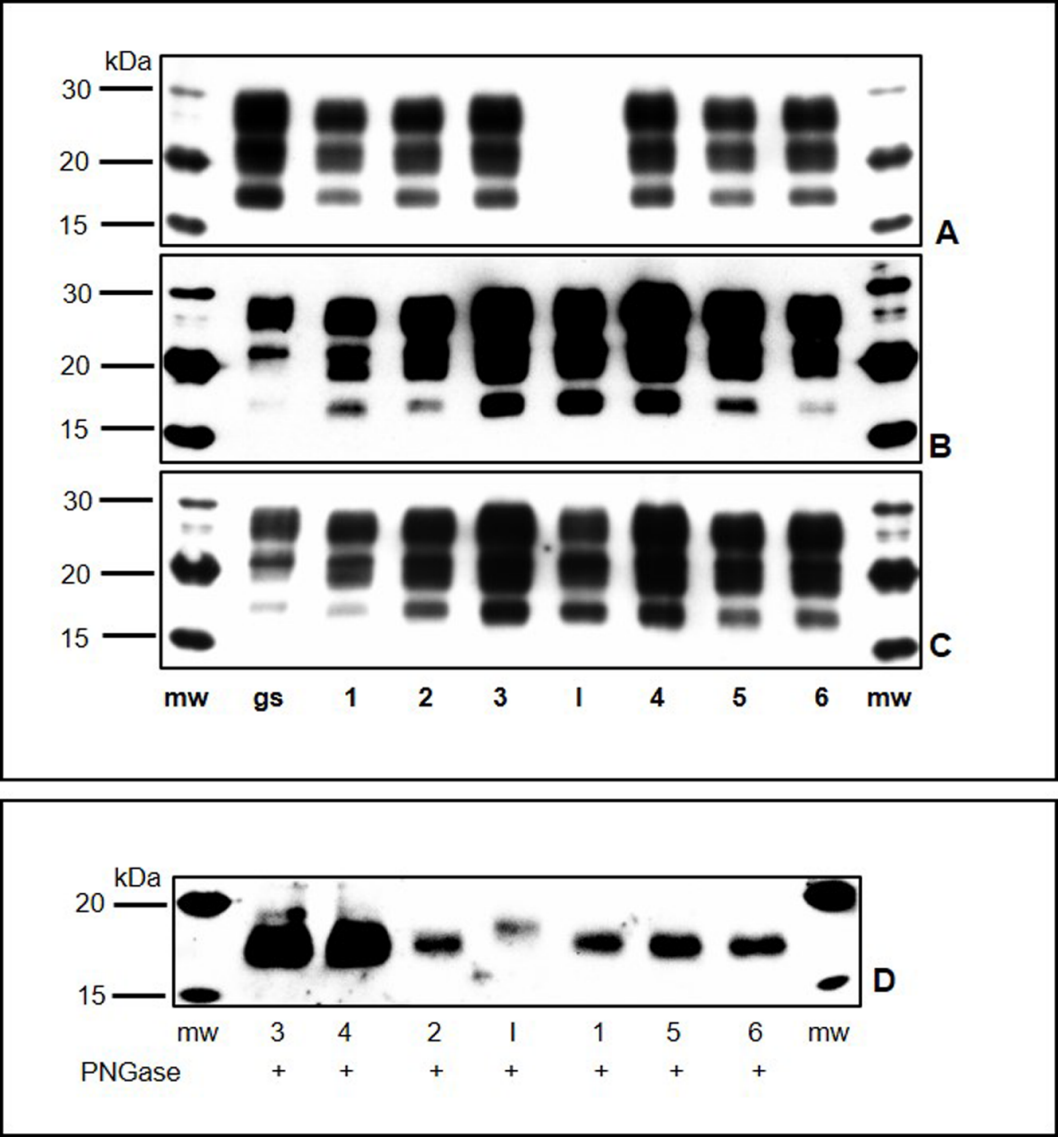
Western blot analysis of proteinase K-treated homogenates from brainstems of goats intracerebrally inoculated with L-BSE. I: L-BSE isolate (141387/02) used for *inoculum*; lanes 1–6: inoculated goats; gs: goat with natural classical scrapie. MW: molecular size markers. Membranes were probed with three different monoclonal antibodies: P4 (A), 6H4 (B), and SAF84 (C). Panel D shows the proteinase K-treated homogenates treated with enzymatic deglycosylation (PNGase) and detected with mAb SAF84. WB analyses of tissue samples from the experimentally challenged goats showed the presence of PrPSc, with an electrophoretic pattern characterized by three bands corresponding to the di-, mono-, and un-glycosylated forms.

Western blot (WB) analyses carried out on the C-BSE and L-BSE isolates used for the inoculum exhibited the same molecular features of PrPSc as described in many previous studies: i) lower molecular mass of the un-glycosylated band of the L-BSE isolate than that of C-BSE; ii) prominent fraction of the mono-glycosylated band in the L-BSE sample and a higher value of the di-glycosylated band in C-BSE; iii) no detection or sometimes very weak signals of PrPSc with mAb P4 immunolabelling (Figs 8 and 9 and S1 Fig).

Preliminary analysis with mAb F99.97.6.1 carried out on the nervous tissue from C-BSE and L-BSE goats, bovine inocula and Scrapie control showed, except for the molecular weight of the unglycosylated band, similar molecular pattern of PrPSc. (S2 Fig). However, unlike the C-BSE and L-BSE bovine isolates, strong PrPSc signals were detected with mAb P4 in both groups of goats, particularly in those infected with C-BSE (Figs 8 and 9, panels A, B, and C and S1 Fig). Moreover, there was a slight difference in the molecular weight of PrPSc between the un-glycosylated band of pathological prion protein in the experimentally inoculated goats and the respective donors; specifically, after enzymatic deglycosylation, the PrPSc molecular masses were slightly lower in the goats infected with C-BSE [17.58 kDa (± 0.08 standard deviation [SD])] and L-BSE [17.53 kDa (± 0.1)] as compared to the C- and L-BSE isolates [19 kDa and 18.5 kDa, respectively] (Figs 8 and 9, panel D). Moreover, the di-glycosylated band in the C-BSE infected goats showed the strongest signal, followed by the mono-glycosylated and the un-glycosylated bands. The L-BSE infected goats displayed a glycoform ratio of PrPSc different from that of the L-BSE bovine isolate, with intermediate values between the C-BSE bovine isolate and the control goat with classical scrapie, especially in relation to the monoclonal antibody used for this purpose. In detail, the mean (± standard deviation [SD]) di- and mono-glycosylated band intensity with mAb 6H4 was 68.10 ± 4.08: 26.78 ± 2.39 but 59.08 ± 1: 34.87 ± 0.95 with mAb SAF84, respectively.

#### PrPSc distribution in peripheral tissues

**Fig 10:**
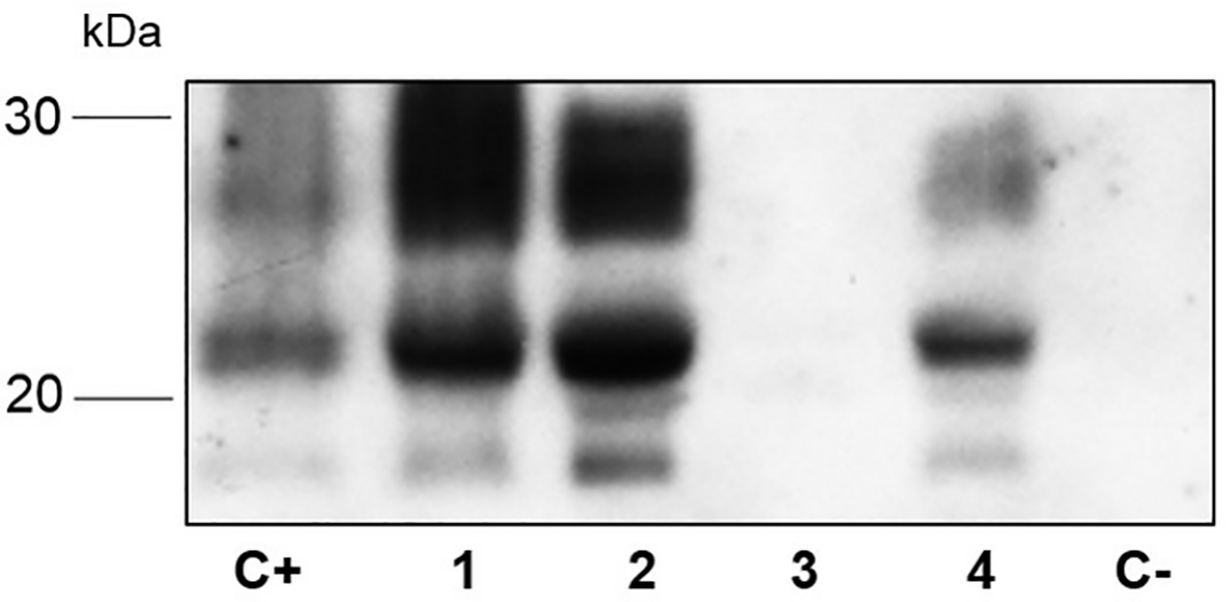
Western blot analysis of proteinase K-treated homogenates from spleen of goats intracerebrally inoculated with C-BSE. C+: spleen sample of goat affected with natural scrapie; C- = spleen sample of healthy goat; Lanes 1–4: spleen samples of goats experimentally infected with BSE [81556 (1), 81559 (2), 69556 (3), 69557 (4)]. Membrane was probed with mAb P4.

PrPSc was detected in the lymphoreticular system (spleen, tonsils, and lymph nodes) of C-BSE positive animals by immunohistochemical analysis. The submandibular (Fig 5G) and retropharyngeal lymph nodes demonstrated strongly staining aggregates in the lymphoid follicles of all the C-BSE goats, whereas parotid lymph nodes, tonsils, ileocecal valve (gut-associated lymphoid tissue [GALT]) (Fig 5H) resulted positive only in 1/4, 2/4, and 2/4 animals, respectively. WB confirmed peripheral positivity in three out of four spleens in the C-BSE goats but not in the other lymphoreticular tissues (Fig 10). No peripheral distribution was detected in the L-BSE goats.

## Discussion

The present study provides the first evidence of successful transmission of L-BSE to goats via the intracerebral route, allowing its comparison with C-BSE infection in goats. The disease phenotypes observed in the C-BSE and L-BSE infected goats showed similar clinical manifestations: early clinical signs were skin lesions mainly on the neck, shoulders, sacral region, hind limbs, and tail, probably resulting from scratching against the walls of the shed or under the trough. Other authors have described the presence of pruritus in experimental small ruminants: Konold *et al*. [14,15] identified a pruritic form of C-BSE in sheep and goats, while Simmons *et al*. [12] described a clinical syndrome characterized by pruritic behaviour in experimental L-BSE sheep that resulted in wool loss and ataxia. A neurological explanation for pruritus in small ruminants with TSEs is difficult to provide. Hadlow hypothesized that pruritus in scrapie affected goats was central and that it originated in the main subcortical sensory nucleus in the thalamus [16]. Our results seem to confirm this hypothesis, since the thalamus in both the C-BSE and the L-BSE group showed a high grade of vacuolation. Noteworthy was lateralization of clinical signs (positional strabismus and positional vertical nystagmus) in three L-BSE goats, suggesting involvement of the vestibular system; histopathological examination of the brain ruled out a secondary disease that could have caused asymmetrical clinical presentation. A vestibular syndrome was described by Konold *et al*. [17] in two goats with classical scrapie and by Acutis *et al*. [18] in experimentally challenged goats carrying mutation K222 of the prion protein gene. It is worrying that many of the clinical features described here are frequently reported in small ruminants with scrapie, confounding the diagnosis of disease under field conditions and emphasizing the utility of histological examination. Moreover, the atypical clinical presentation (vestibular clinical signs) by three animals stresses the importance of not excluding TSEs from differential diagnosis on the basis of lateralization of neurological signs under experimental and field conditions.

Although the attack rate for C-BSE and L-BSE was 100% for both groups, the incubation period (IP) was significantly shorter for the C-BSE group than the L-BSE group (mean IP 568.25 dpi vs. 1081.33 dpi, respectively) and roughly equivalent to that reported by other experimental C-BSE transmission studies in goat species [7,15], given that some variations in time will be due to the different genotypes of animals. The survival time (SV) of the C-BSE goats was also significantly shorter than that of the L-BSE goats (mean SV 624.5 dpi vs. 1239 dpi, respectively), contrary to what has been reported in other experimental transmission studies [19–23]. Furthermore, though the inoculum source was the same (IT 141387/02, 10% w/v) as that used for ARQ/ARQ L-type sheep described by Simmons *et al*. [12], comparison of the incubation periods and the survival times for the two groups indicates a lower species barrier for L-BSE in goats than in sheep. These data, together with the identification of two natural C-BSE cases in goat, suggest that this species could be a better recipient for bovine TSEs than sheep.

Confirmatory testing by histology and immunohistochemistry showed differences between C-BSE and L-BSE, allowing differentiation between the two TSE strains. We observed vacuolation with intense spongiosis of the caudal regions of the brain of the C-BSE infected animals, as previously described in C-BSE affected cattle [19]. In the L-BSE group, the rostral areas showed more severe vacuolation, with a lesion profile very similar to that described for experimental L-BSE affected sheep [12] but quite different from that for experimental L-BSE affected cattle, where the vacuolation scores were higher for the mesencephalon and obex (nucleus of the solitary tract and nucleus of the spinal tract of trigeminal nerve) [19].

Different patterns of PrPSc deposition further documented the divergent biological properties of the two strains. Although all the mAbs tested by immunohistochemistry correctly identified the positive cases, mAb F99/97.6.1 gave the best results in terms of pattern and strain discrimination and characterization. Immunolabelling with this antibody produced a greater variety of PrPSc patterns in the C-BSE than in the L-BSE goats. In the C-BSE goats, the granular and glial types were the most frequent patterns of PrPSc deposition, together with linear, intraneuronal, and intraglial types, whereas the granular pattern was mainly present in the L-BSE goats. A clear distinction between the two strains was evident at the level of the cerebellum: in the molecular layer, the glial pattern in the C-BSE goats showed similarity with classical scrapie, whereas the granular pattern in the molecular layer in the L-BSE goats showed similarity with atypical scrapie. To further characterize the two strains, PrPSc epitope mapping with a panel of different monoclonal antibodies was applied. No differences were observed between C- and L-BSE regarding extra- and intraneuronal immunoreactivity, suggesting that this approach is not useful to distinguish bovine strains in goat. However, differently from scrapie and according to other authors [24–26], no intraneuronal PrPSc immunoreactivity was observed using upstream N-terminal antibodies, thus allowing discrimination between bovine strains and scrapie in goat. Astrocytosis was identified using glial fibrillary acidic protein (GFAP) antibody in positive animals; an increased number of astrocytes is an early molecular marker and a typical reaction that occurs in prion diseases in response to the presence of damaged neurons.

The lymphoreticular involvement observed in all of the C-BSE positive goats argues in favour of a peripheral distribution of PrPSc, as reported in previous studies [9,15]. In classical scrapie, lymphoreticular system involvement precedes PrPSc infection of the peripheral nerves and subsequent spread to the CNS [27,28]. Moreover, PrPSc can accumulate in the tonsils in VRQ/VRQ sheep infected with scrapie more than 1 year before the onset of clinical signs [29]. Although the peripheral distribution of PrPSc could represent a threat for consumers in case of C-BSE in goats, postmortem testing of the lymphoreticular system and nervous tissues may increase diagnostic sensitivity and assist in active surveillance and disease eradication at the herd level. As regards the atypical strain, our data seem to suggest that PrPSc of L-BSE in small ruminants is not characterized by peripheral diffusion. In addition, our data are shared by previous studies that obtained similar results in sheep [10] or found positivity in a single animal in the experimental group [11,12].

The rapid tests included in the study were those approved according to Regulation (EC) No. 999/2001. Previous studies showed that BSE-approved rapid tests can detect atypical BSE in bovines despite evidence of differences in relative analytical sensitivity [30]. There is some concern about the systematic sensitivity of rapid tests for the diagnosis of C- BSE in goats [31]. Laboratory performance of rapid diagnostic systems for the detection of atypical BSE strains in small ruminants has not been studied to date. Here, we report on the diagnostic sensitivity of EU-approved rapid tests for the detection of L-BSE in experimentally infected goats.

If we compare the working principle of these rapid tests, the Bio-Rad assays include a proteinase K (PK) digestion step to unmask cryptic epitopes, whereas the IDEXX HerdChek® BSE-scrapie EIA relies on conformational detection technology using a specific aggregate specific capture ligand on a dextran polymer (Seprion ligand technology, Microsens Biotechnologies, London, UK). The severe effects of PK in digesting atypical PrPSc are well known [32]. This could be the reason for the slightly lower OD values obtained with the Bio-Rad tests. However, the dynamic range of each rapid test, or rather the concentration range of PrPSc that results in a change in response, is a peculiarity of each diagnostic system. The rate of conversion of substrate to coloured product should be proportional to the amount of PrPSc within the well, but there are many limits to this depending on the analyte itself, which tends to aggregate rapidly in solution, and on the combination of methods and materials used with the test kits and the equipment. A gradual stratification of the signal represents a *surplus* value for TSE rapid assays.

The results of this rapid test study endorse the current epidemiological follow-up and interpretation of such BSE forms, should they arise in goats. This means that active surveillance applied to fallen stock and/or healthy slaughtered populations will detect such cases, reassuring the epidemiological conclusions.

WB analysis easily detected the presence of the pathological prion protein in all goats experimentally challenged with C-BSE and L-BSE, also with use of an N-terminal antibody like the mAb P4, a particular characteristic usually associated with classical scrapie. In previous transmission studies of classical BSE agent to sheep, WB analysis revealed lower electrophoretic mobility of the un-glycosylated PrPSc band, associated with a loss of prion protein detection by mAb P4, thus suggesting a cut by PK in the epitope recognized by this antibody [33,34]. This differential detection of the pathological prion protein with mAb P4 represents a very important parameter to discriminate scrapie cases from ovine and bovine BSE.

The detection of PrPSc with the P4 in both groups of experimental goats clearly shows that the epitope recognized by this monoclonal antibody is not affected by PK during the digestion phase; therefore, the lowest molecular weight evidenced by the un-glycosylated PrPSc fragment is likely to be attributed to additional C-terminal truncation of the prion protein when the C- and L-BSE agents are passaged in goats.

Reactivity to this mAb has also been observed in transmission studies of L-BSE [11,12,35] and C-BSE to sheep, although after serial passages in the latter case [36]. It is conceivable that C-BSE and L-BSE agents may overcome the species barrier and convert PrPC into a form more like that of classical scrapie. Although our findings are reassuring for diagnostic purposes and indicate that such cases would be detected as positive during routine active surveillance activities, the question is more complex from a discriminatory point of view. The molecular parameters of PrPSc on which basis different WB tests work to exclude the presence of the BSE agent in small ruminants are: i) electrophoretic mobility of the un-glycosylated band; ii) no detection with mAb P4; and iii) glycoform profile. The results of the present study show that experimentally infected cases can be discriminated from classical scrapie in goats mainly by virtue of the electrophoretic mobility of the un-glycosylated band and partially by the glycoform profile, since this latter parameter is the least robust of discriminatory criteria for this technique [33]. Based on these observations, caution and attention are warranted if WB is the only confirmatory test applied for the discrimination of TSE strains in goats.

In conclusion, this is the first report of experimental transmission of L-BSE in goats. The study demonstrated that goats are susceptible to both the classical and atypical strains of BSE; however, these forms can be easily detected and characterized by instruments currently in use via active and discriminatory surveillance of TSEs in goats, which remains a problem for the Mediterranean countries.

## Materials and methods

### Ethical approval

All procedures involving animals and their care were conducted in conformity with national and international laws and policies (EEC Council Directive 86/609, 63/2010; Italian Legislative Decree 116/92 and 26/2014). The study was approved by the Italian Ministry of Health with authorization number 694/2015-PR of 17^th^ of July 2015.

### Animals and animal care

Twenty, seven-month-old goats were purchased from herds with no record of scrapie cases in the last five years. All were free of neurological signs. Nineteen animals were Saanen breed and one was crossbred. A blood sample was taken from each animal for genetic analysis to exclude subjects carrying mutations that could confer resistance against BSE: L168 and M142 [37,38] or Scrapie (I142M, N146S / D, R154H, R211Q, Q222K). Two groups were formed (Table 5): C-BSE intracerebral inoculated (n = 4) and L-BSE intracerebral inoculated (n = 6). One or two goats per group were challenged with phosphate buffered saline (PBS) and served as controls. During the experiment, the controls were kept together with their respective group in the same room so that they would be exposed to identical environmental conditions.

**Table 5:** Antibodies to PrP used for IHC and WB analysis, indicating the corresponding epitopes.

Antibodies	PrP aa residues
F99*	220–225
P4*	93–99
9A2*	102–104
12B2*	93–97
L42*	144–166
2G11*	153–158
6H4*	156–164
SAF84*	163–173

*mouse monoclonal antibodies

### Caprine PRP gene determination

Genomic DNA was isolated from EDTA-treated blood samples using Thermo Labsystems KingFisher kits (Thermo LabSystems Inc., Beverly, MA, USA). PCR amplification of the entire open reading frame of the PRNP gene was performed according to a previously described protocol [39] using the primers p8(+) (5’- CAGGTTAACGATGGTGAAAAGCCACATAGG-3’) and p9(-) (5’-GGAATTCTATCCTACTATGAGAAAAATGAGG-3’) [40]. PRNP polymorphisms were detected by direct DNA sequencing on both strands of the PCR products by using dye terminator cycle sequencing and an ABI Prism 3130 Genetic Analyser (Applied Biosystems, Carlsbad, CA, USA). Sequencing primers were p8(+), p61(+) (5’-AACCAACATGAAG-CATGTGG-3’), p60(-) (5’-GATAGTAACGGTCCTCA-TAG-3’) and p9(-) [41]. The primers were hybridized to the target PRNP DNA at codons 1–7, 109–116, 147–154 and 249–257, respectively (ovine reference sequence GenBank accession number AJ000739).

### Inocula

Brain homogenates from the brainstem of a C-BSE naturally affected cow (IT-128204/01) and from the frontal cortex of a L-BSE naturally affected cow (IT-141387/02) were diluted in PBS. The inocula were ground in a mechanical grinder and homogenized with normal sterile saline solution to a final concentration of 10% (w/v). A solution of penicillin and streptomycin was added to the inocula prior to use and the homogenate was checked for microbiological sterility. C-BSE and L-BSE inocula were prepared to obtain a comparable amount of PrPSc as assessed by Western blot analysis with the 6H4 anti-PrP monoclonal antibody (Prionics AG, Zurich, Switzerland) (S3 Fig).

### Inoculation of goats

To avoid potential cross-contamination, C-BSE and L-BSE transmission experiments were performed on different days. Prior to inoculation, the animals were kept in the new environment for 1 month for adaptation. Just prior to inoculation, the animals were clinically examined to rule out clinical abnormalities. Intracerebral inoculation was performed as previously described [19, 42] with minor modifications. The animals were anaesthetized with xylazine (0.22 mg/kg, i.m.) combined with ketamine (11 mg/kg, i.m.) and a midline skin incision was made with a 1-mm hole drilled through the calvarium. The inoculum (0.5 mL of 10% w/v brain homogenate) was injected into the midbrain through a 22-gauge needle and the skin incision was closed with a single suture. The inoculated animals were housed together in a bio-safety level 3 containment facility.

### Statistical analysis

The incubation period of TSE in goats was defined as the period between administration of the inoculum and the first manifestation of at least two clinical signs described above; survival time was defined as the period between administration of the inoculum and death of the animal. Differences in survival time and incubation period between the two groups were compared using Kaplan-Meier survival estimates. The difference in the probability was analyzed using the log-rank test for equality of survivor functions. All P values were two-sided, and significance was set at a P value ≤ .05. Statistical analysis was performed using Stata/SE 14.0 (StataCorp, College Station, TX, USA).

### Clinical evaluation

Clinical evaluation comprised daily observation and physical assessment carried out by the animal husbandry staff and the veterinarian. Neurologic examination was performed monthly by a board-certified neurologist. For this purpose, a clinical examination protocol previously used to diagnose scrapie in sheep was applied [43]. The protocol followed the standard procedure for assessing mental status, posture, gait, postural reactions and proprioception, cranial nerves, spinal reflexes, and sensitivity. Sensitivity to external stimuli was evaluated by acoustic response, as described for BSE [44], wherein an animal was considered hyperreactive if it showed an exaggerated response three times in a row. The nibble reflex was considered positive if the animal showed head and neck extension, and chewing movements associated with head and tongue movements after being manually stimulated on the withers and lumbosacral areas. An animal was considered symptomatic (onset of symptoms) if it persistently showed at least two of the following clinical signs: abnormal fleece, abnormal mental status/behaviour, abnormal gait, abnormal postural reaction/proprioception, and positive nibble reflex. Animals with suspicious signs underwent frequent neurological examination; animals that became inappetant or tended toward recumbency were humanely euthanized.

### Tissue sample collection and preparation

After induction of general anaesthesia with propofol (PropoVet®, Abbott Animal Health, North Chicago, IL, USA) administered intravenously (i.v.), the animals were euthanized with i.v. enbutramide/ mebezonium iodide/tetracaine hydrochloride (Tanax®, Intervet Inc. Merck, Summit, NJ, USA). After culling, the whole brain, the entire spinal cord, the lymphoreticular system, internal organs, muscles, and peripheral nervous tissues were sampled. Each sample was divided equally: one half was fixed in 10% buffered formalin or Carnoy’s solution for haematoxylin and eosin (H&E) staining and PrP immunohistochemistry, and the other was frozen at -80°C.

### Rapid test

In order to compare the diagnostic sensitivity of EU-approved rapid tests, *medulla oblongata* samples from 4 C-BSE, 5 L-BSE experimentally infected goats and 2 samples from control animals were tested in parallel with IDEXX HerdChek® BSE-scrapie EIA (HerdChek BSE-scrapie antigen test kit, EIA, IDEXX Laboratories, Westbrook, ME, USA) Short and Ultra Short protocol–small ruminant conjugate, IDEXX HerdChek® BSE-scrapie EIA Short and Ultra Short protocol—bovine conjugate; Bio-Rad TeSeE™ SAP (TeSeE SAP purification-detection test kit, Bio-Rad Laboratories, Marnes-La-Coquette, France) and Bio-Rad TeSeE™ Sheep and Goat™ (TeSeE sheep & goat purification-detection test kit, Bio-Rad Laboratories). Each sample was tested systematically in triplicate. The assays were carried out according to the manufacturers’ instructions. General quality standards for repeatability were observed.

### Pathology and immunohistochemistry

After fixation in 10% formalin, the brain and the brainstem of each goat were coronally cut at the level of obex, medulla, pons, cerebellum, midbrain, diencephalon, and telencephalon. The sections were processed and embedded in paraffin and stained with H&E. Six macro-areas (medulla at obex, rostral medulla, cerebellar vermis, midbrain, thalamus and frontal areas) were further divided into 22 sub-areas for analysis as described by Ligios et al. [45] (1 Dorsal nucleus of the vagus nerve, 2 Nucleus of the hypoglossal nerve, 3 Reticular formation, 4 Midline Raphe, 5 Accessory cuneate nucleus, 6 Olivary nuclei; 7 Vestibular nuclear complex, 8 Cochlear nucleus, 9 Nucleus of the spinal tract of the trigeminal nerve, 10 Midline raphe; 11 Nodulus±granular layer, 12 Nodulus±molecular layer; 13 Central grey matter, 14 Red nucleus, 15 Substantia nigra, 16 Lateral geniculate nucleus; 17 Dorsomedial thalamic nucleus, 18 Ventral thalamic nuclei, 19 Area hypothalamica; 20 Caudate nucleus, 21 Nucleus accumbens, 22 Frontal cortex).

The severity and distribution of vacuolar lesions in each sub-area were graded on a scale from 0 (no vacuolation) to 4 (extensive vacuolation) by semi-quantitative analysis with optical microscopy. The analyses were performed by two different operators unaware of whether the animal had been challenged with C-BSE or L-BSE.

Immunohistochemical (IHC) evaluation of PrPSc deposition patterns and PrP distribution patterns was performed on the same 22 subareas. Five-μm thick sections of each formalin-fixed, paraffin embedded specimen were cut. The slides were dewaxed, rehydrated by routine methods, and then immersed in 98% formic acid for 25 min. After washing in distilled water, the sections were autoclaved for 30 min at 121°C in citrate buffer (pH 6.1). Endogenous peroxidase activity was blocked in 3% hydrogen peroxide for 20 min at room temperature (RT). To block non-specific tissue antigens, the sections were incubated with 5% horse blocking serum for 20 min at RT, and then incubated for 1 hour at RT with a panel of primary monoclonal antibodies (mAbs) including F99/97.6.1 (mouse, isotype IgG_1_; Lot. P120227-001; VMRD, Pullman, WA, USA; 1: 1000 dilution;) [46], L42 (mouse, art. n. R8005, Lot. 741012; R-Biopharm AG, Darmstadt, Germany; 1:250 dilution), 9A2 (mouse, clone 76.9A2, Central Veterinary Institute of Wageningen, Lelystad, the Netherlands; 1:1000 dilution), 12B2 (mouse, clone 76.12B2, batch 051112-PrP-12B2, Central Veterinary Institute of Wageningen; 1:1000 dilution), P4 (mouse, clone P4, R-Biopharm AG; 1:2000 dilution), and 2G11 (mouse, art. n. P01610, isotype IgG2a, Institut Pourquier, Montpellier, France; 1:250 dilution). The corresponding epitopes of each antibody for IHC analysis are listed in Table 5. After rinsing, a biotinylated goat anti-mouse secondary antibody (1:200 dilution; Vector Laboratories, Burlingame, CA, USA) was applied to the tissue sections for 30 min at RT, followed by the avidin-biotin peroxidase complex (PK-4002, Vectastain ABC kit; Vector Laboratories) according to the manufacturer’s protocol. PrPSc immunoreactivity was visualized using 3,3’-diaminobenzidine (Dakocytomation, Carpinteria, CA, USA) as chromogen; the sections were then counterstained with Meyer’s haematoxylin. In case of a positive result, specificity was checked by replacing the primary antibody with normal serum. The relative abundance of IHC immunolabelling in the brain was subjectively graded by examination with light microscopy at 20X. All organs and peripheral tissues taken from each goat were tested by IHC analysis with antibody F99/97.6.1. Immunohistochemistry was also performed on the same brain sections used to investigate prion protein deposition using a polyclonal antibody against GFAP (rabbit, code n. Z0334, Lot 096, Dako, Glostrup, Denmark; 1:1000 dilution).

### PrP distribution in peripheral tissues

Approximately 60 tissue samples of internal organs and muscles were collected from each goat to study the peripheral distribution of PrPSc. One half of each sample was formalin-fixed and paraffin-embedded for further histopathological analysis, and the other half was frozen and stored at -80°C until WB analysis.

### WB analysis

To detect the presence of PrPSc and evaluate its molecular features, WB analyses were carried out on the brainstem and extraneural tissues collected at necropsy from the experimentally challenged goats. Brain tissue from C-BSE and L-BSE samples, used for the inoculum, and previously confirmed classical scrapie goat served as controls. PrP extraction from brain and peripheral tissues and detection of the pathological isoform were performed as previously reported [18]. PrPSc was detected using different mAbs: F99.97.6.1 (mouse; isotype IgG_1_; VMRD, Pullman, WA, USA; 1:1000 dilution), P4 (mouse; R-Biopharm, Darmstadt, Germany; Cat. n. R8007), 6H4 (mouse; Prionics, Switzerland; Cat. n. 01–010), and SAF84 (mouse; Cayman Chemical Co., Ann Arbor, MI, USA; Cat. n. 189775). The corresponding epitopes of each antibody used for WB are listed in Table 5. Immunosignals were revealed with an alkaline phosphatase-conjugated goat anti-mouse IgG, a chemiluminescent substrate, and then visualized on Hyperfilm ECL (Amersham, GE-Healthcare Life Sciences, Little Chalfont, Buckinghamshire, UK) or with a gel documentation analysis system (Uvitec, Cambridge, UK). For quantitative study of the glycoform ratios, chemiluminscent signals corresponding to the three glycoforms of PrP^Sc^ were quantified using Image Lab^TM^ software (Bio-Rad). For deglycosylation, proteinase K-digested samples were deglycosylated with recombinant peptide N-glycosidase F (PNGase F) according to the supplier’s instructions (Roche, Applied Science, Penzberg, Germany), and the immunodetecion was carried out only with mAb SAF84. The molecular weight of the un-glycosylated band of the pathological prion protein was estimated by comparison with the molecular weight standards using Image Lab^TM^ analysis software (Bio-Rad).

## Supporting information

S1 Fig Western blot analysis of proteinase K-treated homogenates from brainstems of C-BSE and L-BSE (BASE) goats. Membranes were probed with mAbs P4 (93–99 aa residues), 6H4 (156–164 aa residues) and SAF84 (163–173 aa residues).
(TIF)

S2 Fig Western blot analysis of proteinase K-treated homogenates from brainstems of C-BSE and L-BSE (BASE) goats. Membrane was probed with monoclonal antibody F99.97.6.1 (220–225 aa residues).
(TIF)

S3 Fig Western blot analysis of proteinase K-treated homogenates (10% w/v) of bovine brain used for goat inocula. C-: negative control; 128204: bovine C-BSE; 141387: bovine L-BSE. Membrane was probed with monoclonal antibody 6H4 (156–164 aa residues).
(TIF)
