# Supplementary figures and images for: Clinical, pathological, and molecular features of classical and L-type atypical-BSE in goats
Source: PLoS One. 2018 May 24;13(5):e0198037. doi: 10.1371/journal.pone.0198037 (PMC5968405; doi:10.1371/journal.pone.0198037)

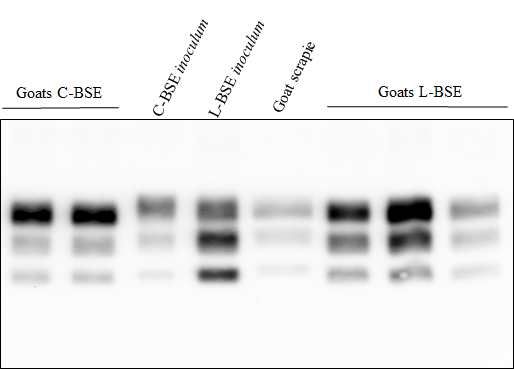

Supplement: S1 Fig — Membranes were probed with mAbs P4 (93–99 aa residues), 6H4 (156–164 aa residues) and SAF84 (163–173 aa residues). (TIF) [file pone.0198037.s001.tif]

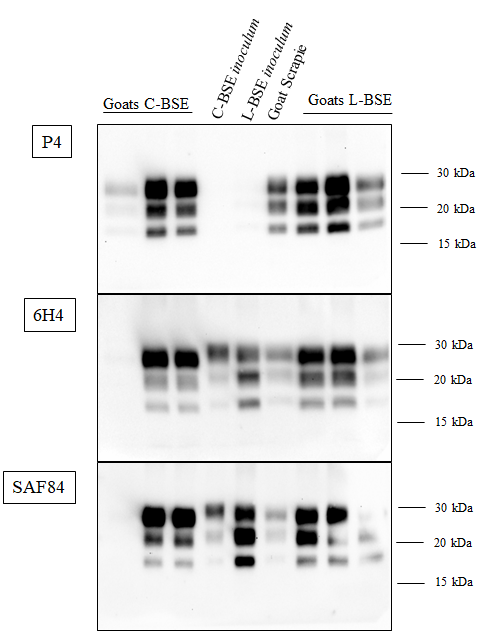

Supplement: S2 Fig — Membrane was probed with monoclonal antibody F99.97.6.1 (220–225 aa residues). (TIF) [file pone.0198037.s002.tif]

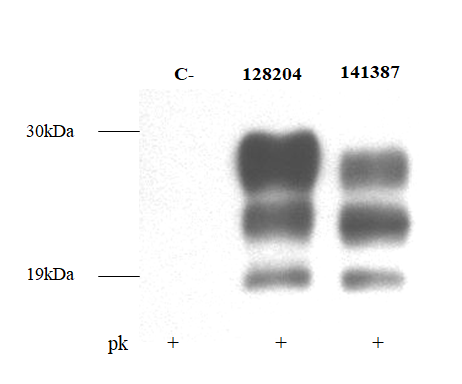

Supplement: S3 Fig — C-: negative control; 128204: bovine C-BSE; 141387: bovine L-BSE. Membrane was probed with monoclonal antibody 6H4 (156–164 aa residues). (TIF) [file pone.0198037.s003.tif]
